# Age-related changes in hematological and biochemical profiles of Wistar rats

**DOI:** 10.1186/s42826-024-00194-7

**Published:** 2024-02-26

**Authors:** Suresh Patel, Satish Patel, Ashvin Kotadiya, Samir Patel, Bhavesh Shrimali, Nikita Joshi, Tushar Patel, Harshida Trivedi, Jitendra Patel, Amit Joharapurkar, Mukul Jain

**Affiliations:** 1 Animal Research Facility, Zydus Research Centre, Zydus Lifesciences Ltd., Ahmedabad, India; 2 Department of Pharmacology and Toxicology, Zydus Research Centre, Zydus Lifesciences Ltd., Ahmedabad, India

**Keywords:** Reference range, Hematology, Biochemistry, Blood, Parameters, Age, Wistar rat

## Abstract

**Background:**

Wistar rats are extensively used as the model for assessing toxicity and efficacy in preclinical research. Hematological and biochemical laboratory data are essential for evaluating specific variations in the physiological and functional profile of a laboratory animal. Establishing hematological and biochemical reference values for Wistar (han) rats at various age intervals was the goal of this work. Male and female Wistar rats (n = 660) of ages 6–8 weeks, 10–14 weeks and > 6 months were used in the experiment. Blood and serum were collected from these rats under fasting conditions.

**Results:**

We observed that the majority of hematological and biochemical parameters were significantly influenced by sex and age. Hematological changes were significantly correlated to aging were increased red blood cells, hemoglobin, hematocrit, neutrophils, monocytes and eosinophils in both sexes, as well as decreased platelet, mean corpuscular volume, mean corpuscular hemoglobin and lymphocytes in both sexes. White blood cells of male rats were considerably higher than those of female rats in all age ranges. For biochemistry, increase in glucose, total protein and creatinine were seen in both sexes, along with increases in urea in females and alanine aminotransferase in males. Age was significantly associated with decreased alkaline phosphatase in both sexes.

**Conclusions:**

When using Wistar rats as a model, these reference values may be useful in evaluating the results.

## Background

Rodents, like mice and rats, are the preclinical animal models of choice in pharmaceutical research. They are especially useful in aging research since they are closely related to humans and mammals and have a relatively small size and a short lifespan, which makes them more feasible to study in comparison to larger and long-lived animals [1]. Most human diseases can be modelled in these rodents by changes in equivalent genes or by physicochemical stimuli [2]. Establishing a specific and sensitive preclinical trial paradigm based on the best rodent models reduces the drug development cost and also minimizes the risk to human subjects in clinical trials [3]. Hence pre-clinical efficacy, toxicity and safety studies in rodents are important in development of new drugs [4]. Wistar rats have been widely used in pharmacology, toxicology, and safety studies [5].

During preclinical research, hematological and biochemical measurements are useful to ascertain the observations made by direct examination of organs and tissues in toxicity and safety studies. Hence, hematologic and biochemical values are critical for assessing the health and disease states associated with the blood disorders, infectious diseases, immune system and lipoprotein metabolism, glucose regulation, and functions of major metabolic organs like liver and kidney. A deviation from the normal range in these parameters can indicate the presence of pathology [6]. Many studies indicate that aging is associated with changes in hematological and biochemical parameters that are indicative of the status of major physiological systems of the body in Wistar rats [7].

Growing age has a significant impact on rodent body weight, and there is a direct relationship between hematological and clinical chemistry parameters including blood volume, cardiac output and stroke volume [8]. Researchers from several nations have reported various reference values for Wistar rats [7, 9–12]. These values can be affected by many factors, such as age, sex, nutrition, animal housing, circadian rhythm, daily activity, stress, sexual cycle etc. Knowledge about the normal hematological and clinical chemistry values in various phases of rat life provides a valuable guide to researchers [13]. Our aim in this study was to contribute to research studies by investigating the hematological and biochemical profiles of Wistar rats reared at Zydus Research Centre under standard conditions. So, using data from years of routine health monitoring of breeding colonies, we have established age-wise reference data of hematology and biochemical parameters for both sexes. These would be a useful reference data set for the evaluation of hematology and clinical chemistry parameters in non-clinical studies.

## Methods

### Animal care and ethical statement

The male and female Wistar (han) rats were bred at Animal Research Facility in Zydus Research Centre. Animals were housed in controlled room temperature of 23 ± 2 °C and humidity conditions of 30–70%, with room ventilation set at 10–15 air changes per hour in IVC (ventilation rate set at 40–50 air changes per hour) with a 12-h light/dark cycle. The animals had access to a standard chow diet (2018 Teklad global 18% protein rodent diets, inotiv) and water ad libitum unless otherwise specified. All the health monitoring procedures complied with CCSEA guidelines and were approved by Institutional Animal Ethics Committee (IAEC). The 6–8 weeks, 10–14 weeks and > 6 months old rats were used in the experiments.

### Selection of animals

As a part of the routine health monitoring program of the breeding colony, randomly selected animals were screened for hematology and biochemistry parameters. The rats were selected from either sexes at 6–8 weeks, 10–14 weeks and > 6 months old breeding colony animals. The data was collected from 660 animals, which includes 110 animals per sex for three age intervals.

### Sample collection

The selected animals were fasted overnight (water ad libitum). Animals were bled by retro-orbital puncture under isoflurane anesthesia. Blood samples were collected in an anticoagulant tube (50 µl/vial, 2% EDTA) and also in an empty tube. The anticoagulant added blood was used for a complete blood count. Then blood collected in an empty tube was allowed to stand for 30 min at room temperature to clot and centrifuged (4000 rpm for 10 min at 24 °C) to harvest serum. The serum samples were used for clinical chemistry analysis.

### Hematology and biochemistry parameter tests

Whole blood was used for determination of hematology parameters: white blood cell (WBC), red blood cell (RBC), hemoglobin (HGB), hematocrit (HCT), mean corpuscular volume (MCV), mean corpuscular hemoglobin (MCH), mean corpuscular hemoglobin concentration (MCHC), platelet count (PLT) and differential WBC count (neutrophils, lymphocytes, eosinophils, monocytes, basophils). The analyses were performed on the automated blood cell analyser CELL-DYN® 3700 System (Abbott) and ADVIA 2120i (Siemens Healthineers, USA). The hematology parameters, their abbreviations, units and measurement method are shown in Table 1.

**Table 1:** Sex specific reference range for hematological parameters in 6–8 weeks Wistar rats

Parameters	Male				Female			
	n	Mean ± SD	Median	Reference range	n	Mean ± SD	Median	Reference range
WBC^a^ (10^3^/µL)	106	5.38 ± 1.62^*^	5.10	2.45–9.55	104	4.89 ± 1.38	4.49	2.88–8.17
RBC (10^6^/µL)	107	6.61 ± 0.38	6.62	5.86–7.35	103	6.85 ± 0.38^**^	6.82	5.97–7.69
HGB (g/dL)	108	13.14 ± 0.78	13.20	11.57–14.63	105	13.48 ± 0.6^**^	13.50	12.10–14.70
HCT (%)	109	41.79 ± 3.01	41.30	35.85–47.75	105	42.80 ± 2.56^*^	42.90	37.57–48.10
MCV^a^ (fL)	108	63.48 ± 3.21	63.95	55.41–68.58	102	62.76 ± 3.14	63.25	55.37–68.27
MCH^a^ (pg)	108	19.89 ± 0.89^*^	20.00	17.96–22.03	100	19.65 ± 0.77	19.50	18.25–21.55
MCHC^a^ (g/dL)	109	31.42 ± 1.17	31.50	29.38–33.83	104	31.51 ± 1.34	31.45	29.49–34.35
PLT (10^3^/µl)	99	757.17 ± 120.76	762.0	467.5–1004.5	99	765.31 ± 122.70	754.0	562.5–1009.0
NEU^a^ %	106	9.15 ± 2.86	8.80	4.27–15.59	101	9.66 ± 2.93	9.40	4.61–15.05
LYMPH %	107	85.39 ± 3.88	85.50	76.84–92.01	100	84.88 ± 3.62	84.85	78.21–92.14
MONO %	104	2.63 ± 1.5	2.41	0.21–5.99	104	2.86 ± 1.64	2.98	0.32–7.54
EOS^a^ %	104	0.76 ± 0.33	0.69	0.25–1.72	101	1.02 ± 0.45^**^	0.94	0.34–2.08
BASO^a^ %	109	1.73 ± 1.14	1.64	0.1–4.17	106	1.48 ± 0.95	1.41	0.10–3.47

*Significant parameters at p < 0.05; **Statistically significant parameters at p < 0.001

^a^statistical comparison based on nonparametric test

Serum samples were used for biochemistry parameters: glucose (GLU), aspartate aminotransferase (AST), alanine aminotransferase (ALT), alkaline phosphate (ALP), total bilirubin (TBIL), total protein (TP), albumin (ALB), urea, creatinine (CREA). The analyses were performed using a Cobas C311 analyser (Roche Diagnostics, Switzerland). The biochemistry parameters, their abbreviations, units and measurement method are summarized in Table 1.

### Statistical analysis

The values for each of the reported parameters were grouped by sex and age. Individual histograms for each hematology and biochemistry parameter in each group were visually checked for outliers, and extreme values were handled according to the D/R ratio [14, 15]. After removing significant outliers, the Kolmogorov–Smirnov test was used to assess the normality of the data distribution for all three age intervals. Reference ranges have been calculated by determinations of the 2.5th and 97.5th percentiles, which include both sexes according to age intervals. All calculations were performed in accordance with the CLSI and ASVCP guidelines [16, 17]. Based on the data distribution, the effect of gender was compared using the independent-sample t-test and Mann–Whitney U test using a statistical software program (SPSS 21.0). The differences linked to age were performed by one-way ANOVA (post- hoc analysis using Tukey HDS test) using a statistical software program (SPSS 21.0). *p* value < 0.05 was considered statistically significant. The data is presented as mean, standard deviation and median.

## Results

### Effect of age and sex on hematological parameters

**Fig. 1:**
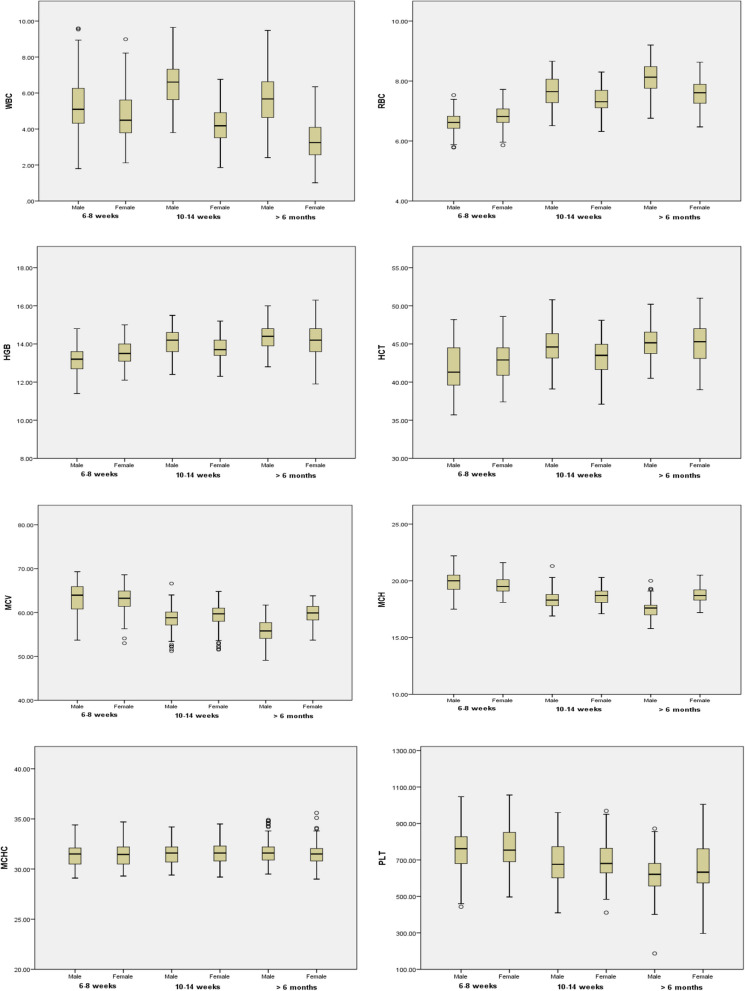

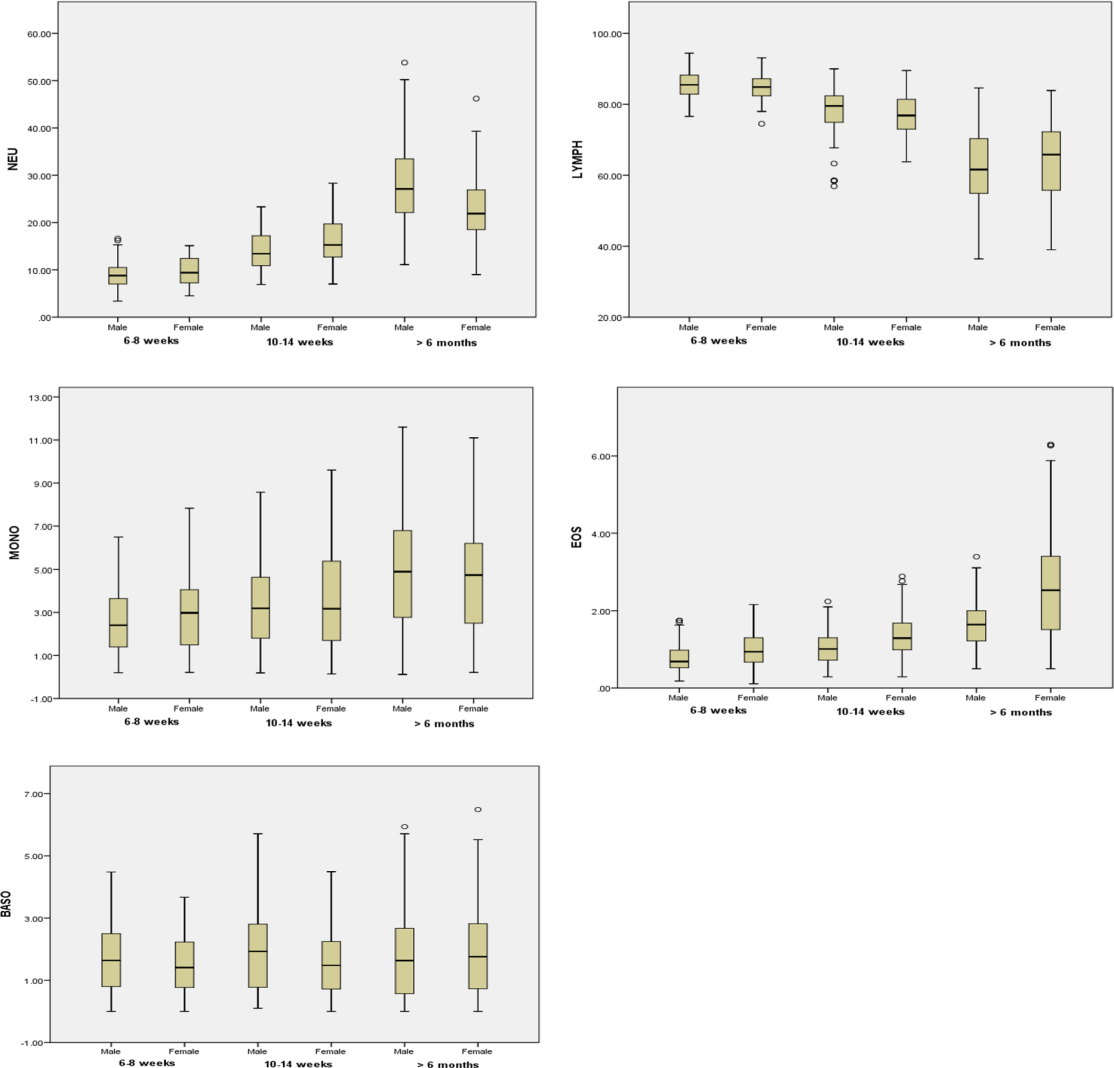
Box plots show differences in hematology parameters of male and female Wistar rats at different ages

Sex differences for 6–8 weeks old rats are summarized in Table 2 as mean, SD, median, and reference range. Male rats had significantly higher WBC and MCH compared to female rats. Female rats had significantly higher RBC, HGB, HCT, and EOS% compared to male rats (Fig. 1). No significant differences between male and female rats were found in MCV, MCHC, PLT, NEU%, LYMPH%, MONO% and BASO% values. In 10–14 weeks old Wistar rats data are summarized in Table 3 as mean, SD, median and reference range. Male rats had significantly higher WBC, RBC, HGB, HCT and BASO% compared to female rats. Female rats had significantly higher MCV, MCH, NEU% and EOS% compared to male rats (Fig. 1). No significant differences between male and female rats were found in MCHC, PLT, LYMPH% and MONO% values. The hematological data of Wistar rats of more than 6 months old rats are summarized in Table 4 as mean, SD, median and reference range. Male rats had significantly higher WBC, RBC and NEU% compared to female rats. Female rats had significantly higher MCV, MCH, PLT and EOS% compared to male rats (Fig. 1). No significant differences between male and female rats were found in HGB, HCT, MCHC, LYMPH%, MONO% and BASO% values.

**Table 2:** Sex specific reference range for hematological parameters in 10–14 weeks Wistar rats

Parameters	Male				Female			
	n	Mean ± SD	Median	Reference range	n	Mean ± SD	Median	Reference range
WBC (10^3^/µL)	101	6.50 ± 1.32^**^	6.61	4.03–9.50	103	4.20 ± 1.07	4.18	2.23–6.50
RBC (10^6^/µL)	104	7.67 ± 0.48^**^	7.65	6.73–8.57	107	7.36 ± 0.43	7.31	6.42–8.21
HGB^a^ (g/dL)	104	14.15 ± 0.68^**^	14.20	12.7–15.38	105	13.76 ± 0.64	13.70	12.50–15.14
HCT (%)	103	44.76 ± 2.36^**^	44.60	40.24–49.38	107	43.27 ± 2.47	43.50	37.38–47.89
MCV^a^ (fL)	95	58.40 ± 2.81	58.80	51.84–63.96	103	59.19 ± 2.73^*^	59.70	51.94–63.70
MCH^a^ (pg)	99	18.35 ± 0.76	18.30	17.05–20.15	106	18.65 ± 0.70^*^	18.70	17.27–20.20
MCHC (g/dL)	102	31.62 ± 1.07	31.60	29.76 – 34.00	103	31.63 ± 1.17	31.60	29.60–34.38
PLT (10^3^/µl)	97	688.99 ± 116.31	676.0	463.95–947.4	99	692.68 ± 108.0	681.0	487.0–951.0
NEU^a^ %	98	14.09 ± 3.68	13.40	7.75–20.71	106	16.01 ± 4.82^*^	15.25	7.90–26.99
LYMPH^a^ %	100	78.34 ± 6.59	79.55	58.40–88.15	106	77.1 ± 5.92	76.85	65.50–89.03
MONO %	103	3.38 ± 2.1	3.19	0.22–7.75	107	3.61 ± 2.26	3.17	0.32–8.24
EOS %	96	1.06 ± 0.44	1.01	0.33–2.089	99	1.36 ± 0.55^**^	1.29	0.51–2.73
BASO^a^ %	103	1.88 ± 1.29	1.93	0.1–5.01	107	1.55 ± 1.07	1.48	0.07–3.77

*Significant parameters at p < 0.05; ^**^ Statistically significant parameters at *p* < 0.001

^a^Statistical comparison based on nonparametric test

**Table 3:** Sex specific reference range for hematological parameters in > 6 months Wistar rats

Parameters	Male				Female			
	n	Mean ± SD	Median	Reference range	n	Mean ± SD	Median	Reference range
WBC (10^3^/µL)	109	5.68 ± 1.28^**^	5.67	3.06–8.51	106	3.34 ± 1.02	3.25	1.80–6.03
RBC (10^6^/µL)	109	8.16 ± 0.5^**^	8.13	7.21–9.12	106	7.61 ± 0.46	7.61	6.71–8.62
HGB (g/dL)	109	14.36 ± 0.64	14.40	12.80–15.80	107	14.27 ± 0.87	14.20	12.64–16.06
HCT (%)	104	45.10 ± 2.16	45.15	40.50–49.81	107	45.05 ± 2.88	45.30	39.34–50.83
MCV^a^ (fL)	105	55.74 ± 2.7	55.80	49.20–60.61	102	59.66 ± 2.39^**^	59.90	53.87–63.43
MCH^a^ (pg)	104	17.53 ± 0.74	17.60	16.13–19.30	106	18.77 ± 0.68^**^	18.70	17.60–20.23
MCHC^a^ (g/dL)	104	31.68 ± 1.19	31.60	29.56–34.80	103	31.60 ± 1.19	31.50	29.50–34.50
PLT^a^ (10^3^/µl)	97	618.96 ± 107.39	621.00	412.25–849.25	98	665.50 ± 141.79	633.00	377.63–963.83
NEU %	107	28.13 ± 8.07^**^	27.10	14.82–47.40	97	23.17 ± 7.31	21.90	9.80–39.21
LYMPH^a^ %	109	62.11 ± 10.35	61.60	40.08–79.25	107	63.73 ± 11.22	65.80	40.20–83.27
MONO %	108	4.88 ± 2.64	4.90	0.19–10.98	100	4.43 ± 2.42	4.74	0.38–9.96
EOS^a^ %	98	1.64 ± 0.59	1.64	0.70–3.09	101	2.68 ± 1.44^**^	2.53	0.62–6.27
BASO^a^ %	102	1.81 ± 1.45	1.635	0–5.58	97	1.95 ± 1.55	1.76	0–5.49

*Significant parameters at *p* < 0.05; **Statistically significant parameters at *p* < 0.001

^a^statistical comparison based on nonparametric test

**Table 4:** Sex specific reference range for Biochemical parameters in 6–8 weeks Wistar rats

Parameters	Male				Female			
	n	Mean ± SD	Median	Reference range	n	Mean ± SD	Median	Reference range
GLU (mg/dL)	108	50.37 ± 16.84	51.20	16.64–85.76	104	64.27 ± 17.07^**^	63.65	34.43–104.46
AST^a^ (U/L)	103	145.52 ± 32.67	141.70	94.34–228.28	109	147.73 ± 40.61	140.60	82.53–230.75
ALT^a^ (U/L)	109	32.66 ± 8.25^**^	31.30	19.78–50.55	110	27.99 ± 5.78	28.50	17.79–39.53
ALP^a^ (U/L)	108	259.17 ± 79.37^**^	242.00	137.35–437.41	105	143.26 ± 44.21	130.40	70.89–250.46
TBIL (mg/dL)	87	0.16 ± 0.07	0.15	0.02–0.31	85	0.15 ± 0.06	0.15	0.05–0.26
TP^a^ (g/dL)	108	5.96 ± 0.31	6.00	5.27–6.53	106	6.18 ± 0.3^**^	6.15	5.57–6.73
ALB^a^ (g/dL)	110	3.79 ± 0.41	3.60	3.2–4.62	110	4.02 ± 0.44^**^	3.80	3.38–4.9
UREA (mg/dL)	100	28.43 ± 7.25	28.55	13.57–42.56	97	39.15 ± 9.31^**^	39.40	21.75–58.81
CREA^a^ (mg/dL)	110	0.45 ± 0.15	0.51	0.21–0.68	110	0.51 ± 0.15^**^	0.56	0.24–0.75

*Significant parameters at *p* < 0.05; **Statistically significant parameters at *p* < 0.001

^a^ statistical comparison based on nonparametric test

**Fig. 2:**
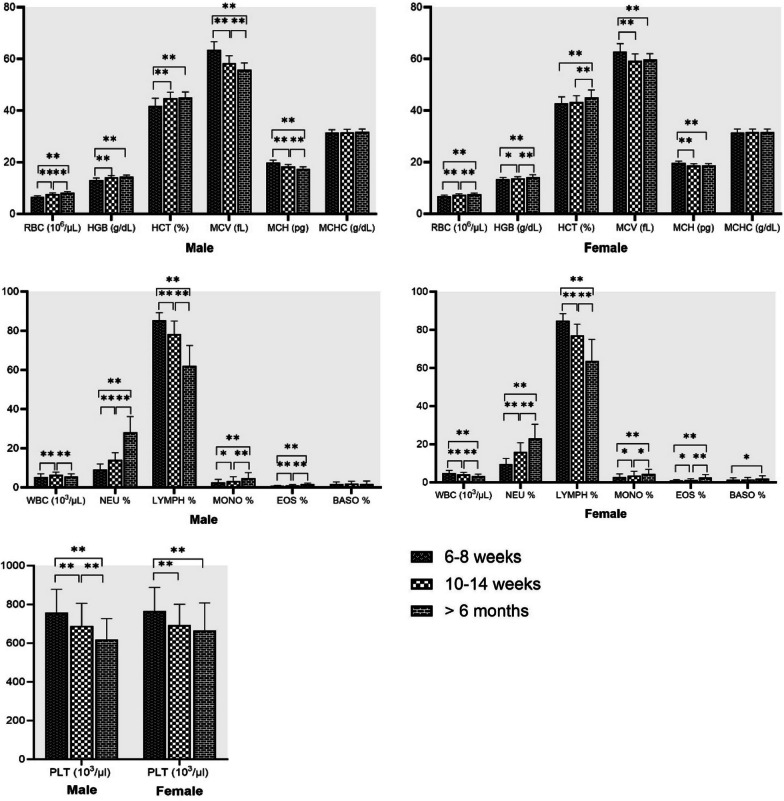
Age related hematological mean values in male and female Wistar rat. Statistical significant differences among values observed in the three age interval (6–8 weeks, 10–14 weeks and > 6 months) are also indicated: *p < 0.05, **p < 0.001

Age-related changes in hematological parameters are presented in Fig. 2 for both sexes. MCHC and BASO% were similar between age intervals and sex; however, BASO% was significantly higher in females of > 6 months old rats than that in rats aged 6–8 weeks and 10–14 weeks. The differential leucocyte counts showed a higher percentage of LYMPH than NEU which is characteristic of rats [18, 19]. RBC, HGB, HCT, NEU%, MONO% and EOS% were significantly higher in > 6 months old rats of both sexes when compared with rats aged 6–8 weeks and 10–14 weeks. PLT and LYMPH% was significantly higher in 6–8 weeks old rats of both sexes when compared with rats aged 10–14 weeks and > 6 months. In addition, 10–14 weeks male rats had significantly higher WBC and 6–8 weeks old female rats had higher WBC when compared with other age groups.

### Effect of age and sex on biochemical parameters

**Fig. 3:**
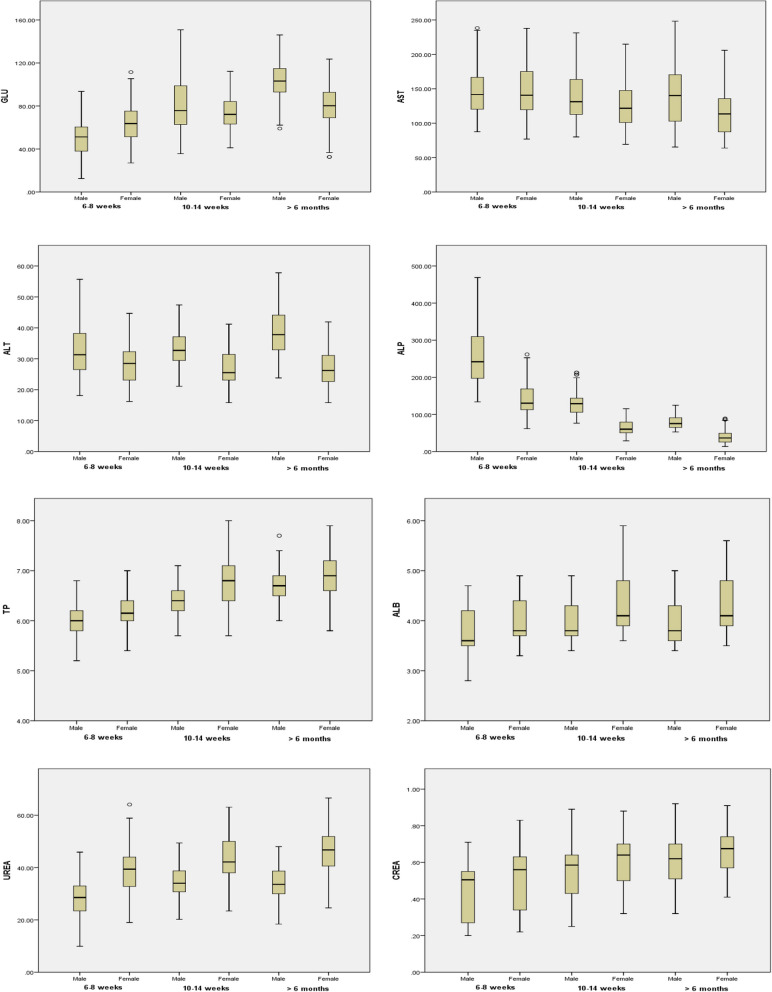
Box plots show differences in biochemical parameters of male and female Wistar rats at different ages

Male rats (6–8 weeks old) had significantly higher ALT and ALP compared to female rats. Female rats had significantly higher GLU, TP, ALB, UREA, and CREA compared to male rats (Fig. 3). No significant differences between male and female rats were found in AST values. The data for 6–8 weeks old male and female rats are summarized in Table 5 as mean, SD, median, and reference range. Male rats (10–14 weeks old) had significantly higher GLU, AST, ALT, and ALP compared to female rats. Female rats had significantly higher TP, ALB, UREA, and CREA compared to male rats (Fig. 3). The data for 10–14 weeks old male and female rats are presented in Table 6 as mean, SD, median, and reference range. In age group of more than 6 months old rats, biochemical parameter values of both sexes are depicted in Table 7 as mean, SD, median and reference range. Male rats had significantly higher GLU, AST, ALT and ALP compared to female rats. Female rats had significantly higher TP, ALB, UREA and CREA compared to male rats (Fig. 3).

**Table 5:** Sex specific reference range for Biochemical parameters in 10–14 weeks Wistar rats

Parameters	Male				Female			
	n	Mean ± SD	Median	Reference range	n	Mean ± SD	Median	Reference range
GLU (mg/dL)	107	80.61 ± 25.85^*^	75.70	39.55–137.06	104	73.64 ± 15.93	72.20	43.48–111.18
AST^a^ (U/L)	106	139.51 ± 35.21^*^	131.25	85.71–213.33	104	125.6 ± 32.56	121.70	72.94–204.13
ALT^a^ (U/L)	107	33.38 ± 5.48^**^	32.70	22.68–45.64	109	26.63 ± 5.49	25.50	16.53–37.95
ALP^a^ (U/L)	102	129.13 ± 30.8^**^	129.00	81.16–209.65	106	64.82 ± 18.00	60.30	36.47–108.52
TBIL (mg/dL)	90	0.14 ± 0.08	0.15	0.02–0.42	91	0.16 ± 0.06	0.15	0.05–0.29
TP^a^ (g/dL)	103	6.39 ± 0.31	6.40	5.76–6.94	110	6.77 ± 0.46^**^	6.80	5.78–7.9
ALB^a^ (g/dL)	108	3.96 ± 0.4	3.80	3.4–4.8	110	4.35 ± 0.55^**^	4.10	3.7–5.6
UREA (mg/dL)	95	34.52 ± 6.14	34.00	21.74–48.2	98	43.41 ± 8.78^**^	42.15	23.75–60.74
CREA^a^ (mg/dL)	108	0.55 ± 0.13	0.59	0.3–0.78	110	0.61 ± 0.13^*^	0.64	0.35–0.87

*Significant parameters at *p* < 0.05; **Statistically significant parameters at *p* < 0.001

^a^Statistical comparison based on nonparametric test

**Table 6:** Sex specific reference range for Biochemical parameters in > 6 months Wistar rats

Parameters	Male				Female			
	n	Mean ± SD	Median	Reference range	n	Mean ± SD	Median	Reference range
GLU (mg/dL)	106	103.37 ± 18.67^**^	103.20	63.75–143.63	108	80.42 ± 20.32	80.20	35.57–122.99
AST^a^ (U/L)	109	138.84 ± 41.78^**^	140.20	75.2–215.58	109	114.81 ± 31.59	113.50	67.33–198.43
ALT (U/L)	102	38.75 ± 7.72^**^	37.80	25.65–54.16	99	26.92 ± 5.66	26.20	17.05–40.95
ALP^a^ (U/L)	109	79.24 ± 17.98^**^	75.40	54.23–117.5	103	38.44 ± 17.22	36.50	14.34–86.4
TBIL (mg/dL)	92	0.13 ± 0.07	0.15	0.02–0.28	82	0.16 ± 0.04	0.16	0.08–0.24
TP^a^ (g/dL)	106	6.73 ± 0.36	6.70	6.07–7.4	109	6.94 ± 0.43^**^	6.90	6.16–7.83
ALB^a^ (g/dL)	110	3.97 ± 0.45	3.80	3.48–4.9	110	4.28 ± 0.55^**^	4.10	3.58–5.4
UREA (mg/dL)	99	34.09 ± 6.43	33.60	20.5–46.7	98	46.31 ± 8.03^**^	46.75	29.85–63.16
CREA^a^ (mg/dL)	110	0.60 ± 0.14	0.62	0.34–0.9	108	0.66 ± 0.12^*^	0.68	0.41–0.89

*Significant parameters at *p* < 0.05; **Statistically significant parameters at *p* < 0.001

^a^Statistical comparison based on nonparametric test

**Table 7:** Abbreviations, units of measurements and methods of analysis of different hematological and biochemical parameters

Parameters	Abbreviations	Units	Method of analysis
Total leukocyte count	WBC	10^3^/μL	Laser light scatter
Erythrocyte count	RBC	10^6^/μL	Light scattering—Optical Cytometer
Hemoglobin concentration	HGB	g/dL	Cyanide-free hemoglobin Methods
Hematocrit	HCT	%	calculated
Mean corpuscular volume	MCV	fL	Cumulative pulse Height Detection
Mean corpuscular hemoglobin	MCH	Pg	Calculated
Mean corpuscular hemoglobin concentration	MCHC	g/dl	Calculated
Platelet	PLT	10^3^ /μL	Light Scattering—Optical Cytometer
Neutrophil	NEU	%	Flow cytometry
Lymphocyte	LYMP	%	Flow cytometry
Monocyte	MONO	%	Flow cytometry
Eosinophil	EOS	%	Flow cytometry
Basophil	BASO	%	Flow cytometry
Glucose	GLU	mg/dL	Hexokinase method
Aspartate aminotransferase	AST	U/L	IFCC method
Alanine aminotransferase	ALT	U/L	
Alkaline phosphatase	ALP	U/L	
Total bilirubin	TBIL	mg/dL	Colorimetric Diazo method
Total protein	TP	g/dL	Colorimetric Biuret method
Albumin	ALB	g/dL	Bromocresol Green method
Urea	UREA	mg/dL	Kinetic method
Creatinine	CREA	mg/dL	Jaffe method

**Fig. 4:**
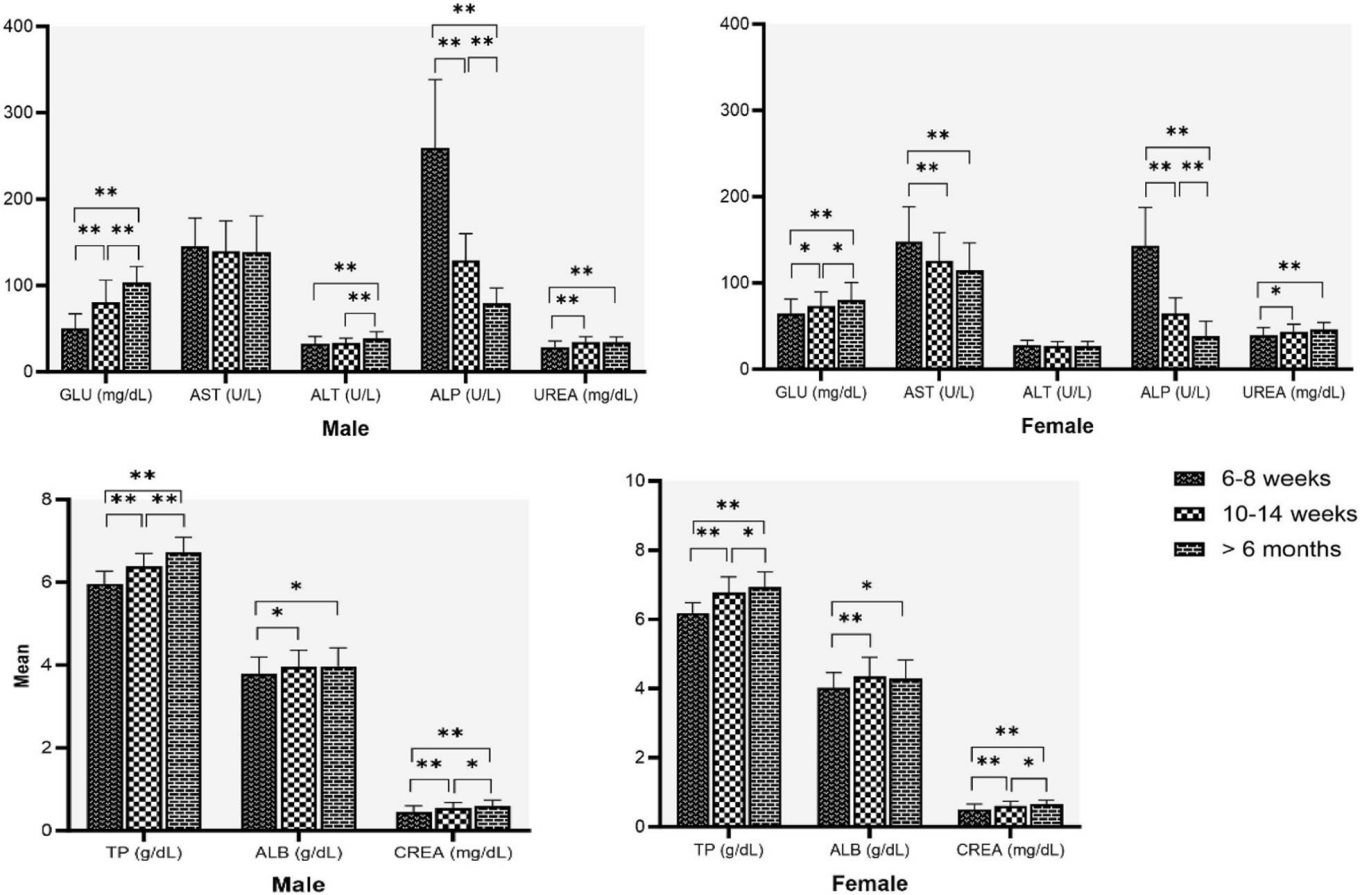
Age related biochemical mean values in male and female Wistar rat. Statistical significant differences among values observed in the three age interval (6–8 weeks, 10–14 weeks and > 6 months) are also indicated: *p < 0.05, **p < 0.001

Age differences for all rats in biochemical parameters are presented in Fig. 4 for both sexes. AST, ALT and ALP showed a significant gender difference and were found higher in male rats, except for AST in 6–8 weeks old rats. TP, ALB, UREA and CREA also showed a gender difference and were found higher in female rats. GLU was found to be significantly higher in females of aged 6–8 weeks, but it was significantly higher in male rats aged 10–14 weeks and > 6 months. GLU, TP, ALB, UREA and CREA significantly higher in > 6 months old rats when compared with rats aged 6–8 weeks and 10–14 weeks. ALP was significantly higher in 6–8 weeks-old rats when compared with rats aged 10–14 weeks and > 6 months. In addition, AST was found to be significantly higher in females of 6–8 weeks when compared with other age groups. TBIL levels did not reach the detection limit in most animals and were not subjected to further statistical analysis.

## Discussion

Knowing the right age of the animals in the experiments improves the reliability and reproducibility of the experiments. It also reduces the number of animals by reducing variability. Choosing the appropriate age of the animals may also ensure the correct population of animals corresponds to humans [20]. The screening of the disease, which requires a specific age of population, also warrants the use of animals of the correct age. In certain diseases, such as anemia of ageing or polycystic ovarian syndrome or post-menopausal diseases, old aged animals are of specific use [21]. The establishment of age-related historical data is required for the laboratory. Thus, we have characterized the Wistar rat data from 6 to 8 weeks to more than 6 months of age, the age group that is mostly used in experiments with male and female rats.

Hematology and biochemistry data determine the effect of drugs without direct examination of organs and tissues for toxicity. Thus, critical assessment of reference values is associated with the diagnosis of disease and organ function. Several studies have shown the values of physiological, biochemical, hematological parameters in rats [7, 9–12, 22–25]. We have observed that RBC, HGB, HCT increase as age increases irrespective of sex differences. Similar observations were also reported in previous studies [11, 23, 25]. It might be due to the effect of testosterone, which activates erythropoiesis by stimulating erythropoietin production. The MCV and MCH decrease with increasing age without sex differences. The reported values were similar to the results reported by Jacob et al. [11]. The MCHC remains unaltered in the entire age group and sex.

It is reported that aging causes an increase in bleeding time and a decrease in PLT count [26]. Similar finding was observed in our experiments showing a decrease in PLT count with increasing age. We also observed a higher PLT count in females than males, a similar finding reported in humans [27, 28]. Males showed a significantly higher WBC than females. WBC progressively decreases in females with age, which is not observed in males. NEU%, MONO% and EOS% values increased with age, whereas LYMPH% decreased with age in both sexes. The findings were in agreement with studies in Wistar rats [11, 12, 23].

Biochemical parameters exhibited significant gender differences in Wistar rats. The GLU, AST, ALT and ALP values were higher in male rats, while female rats had a higher level of TP, ALB, UREA and CREA which matched the reported data [22, 29]. ALP, TP and ALB showed differences relating to both age and sex. ALP decreases with age in both sexes, while TP and ALB increase with age in both sexes [30, 31]. The decrease in ALP may be related to reduced bone health and increased anemia as age increases. The ALP is lower in females, whereas TP and ALB are higher in females than in males in all aged rats. AST and ALT are biomarkers for liver function. Only females showed an age-related decrease in AST. Glucose is a metabolic marker for insulin resistance. In humans and rats, males are more prone to develop age-related diabetes than females [32, 33]. We also observed a similar increase in glucose as age increased, and males had a higher increase in glucose than females as age increased. Kidney function starts deteriorating as age increases. Creatinine and urea in serum are the markers for kidney function. We observed an age-related increase in creatinine and urea in both sexes.

It was challenging to determine an appropriate reference range for selected parameters because of inexplicable outliers without clinical symptoms or outliers with methodological problems. The outlier test was used to remove outliers from the data set, and the majority of the values were found to be within acceptable limits. The fact that we determined reference ranges for a particular strain population of rats with specified environmental factors presents limits to our investigation. Additional research in this area, such as establishing reference ranges with various environmental conditions, might yield more precise data.

## Conclusions

It is evident that the measured hematological and biochemical parameters of Wistar rats can be affected by different factors/conditions. In the present study, we have presented the normal hematological and biochemical parameters of healthy Wistar rats of both sexes at three different age intervals. Moreover, age and sex variations were noted in hematological and biochemical parameters, as well as the lack of these effects in certain parameters. These reference values and age-related values would be useful in studies of aging-related disorders, safety pharmacology or toxicology studies using Wistar rat as a model, as well as to reduce to some extent the number of rats in the control group of future research projects.

## Data Availability

All pertinent information is contained in the manuscript, and the corresponding author can provide original and derived data that support the findings of this work upon request by emailing suresh.g.patel@zyduslife.com.

## Abbreviations

CCSEA: The committee for the control and supervision of experiments on animals
EDTA: Dipotassium ethylenediaminetetraacetic acid
IAEC: Institutional Animal Ethics Committee
